# Going, Going, Gone: Is Animal Migration Disappearing

**DOI:** 10.1371/journal.pbio.0060188

**Published:** 2008-07-29

**Authors:** David S Wilcove, Martin Wikelski

## Abstract

Many of the world's migratory animals are in decline. This essay explores the unique scientific and political challenges of protecting migratory species while they are still common.

Animal migration surely ranks as one of nature's most visible and widespread phenomena. Every minute of every day, somewhere, some place, animals are on the move. The migrants span the animal kingdom, from whales and warblers to dragonflies and salamanders. But is migration an endangered phenomenon? Around the world, many of the most spectacular migrations have either disappeared due to human activities or are in steep decline. Those of us living in eastern North America can no longer experience the flocks of millions of passenger pigeons that temporarily obscured the sun as they migrated to and from their breeding grounds. Nor can residents of the Great Plains climb to the top of a hill and gaze down up hundreds of thousands of bison trekking across the prairies, as was possible less than two centuries ago.

Even the less iconic migrations show signs of trouble. Birdwatchers in North America and Europe, for example, complain that fewer songbirds are returning each spring from their winter quarters in Latin America and Africa, respectively. Indeed, a recent continent-wide analysis of European breeding birds concluded that long-distance migrants (i.e., those species that breed in Europe but winter in sub-Saharan Africa) have suffered sustained and often severe population declines, more so than related nonmigratory species [1]. In central Asia, the number of saiga, a peculiar migratory antelope of the dry steppe grasslands and semi-desert, has dropped by over 95% in the past two decades, from over one million to fewer than 50,000 [2].

The causes of all these declines vary depending on the species and the locale, but in general, the threats to migrants fall into four nonexclusive categories: habitat destruction, the creation of obstacles and barriers such as dams and fences, overexploitation, and climate change. Most of the migrants are in little immediate danger of extinction; rather, they are becoming less and less common. Thus, birdwatchers can still see all of the species of migratory songbirds they seek each spring; they simply have to work harder to do so. Bison still roam national parks and private ranches in the American West, but today's herds number in the hundreds or low thousands, rather than the hundreds of thousands or millions. And there are still lots of salmon to catch off the coast of Norway or British Columbia—just not as many as there used to be.

The question thus arises: Given the panoply of environmental problems we now face, is the fading glory of migration really a significant issue? We would argue that it is. Protecting the abundance of migrants is the key to protecting the ecological importance of migration. As the number of migrants declines, so too do many of the most important ecological properties and services associated with them.

Consider the case of salmon in the Pacific Northwest. The seven species of salmon and seagoing trout in this region share a similar life history strategy: as young fish (smolt), they leave their natal rivers and head to the sea where, aided by the productivity of the ocean, they increase tremendously in size and weight. After a year or two at sea, they return to their natal rivers to spawn, whereupon they die. By migrating upstream, spawning, and dying, they transfer nutrients from the ocean to the rivers. A portion of the nutrients is delivered in the form of feces, sperm, and eggs from the living fish; much more comes from the decaying carcasses of the adults. Phosphorus and nitrogen from salmon carcasses enhance the growth of phytoplankton and zooplankton in the rivers, which provide food for smaller fish, including young salmon. Thus, salmon fry are literally sustained by their parents.

Prior to European settlement, 160–226 million kilograms of salmon migrated each year up the rivers of Washington, Idaho, Oregon, and California. Today, after decades of dam construction, overfishing, water withdrawals for irrigation, logging, and streamside grazing by livestock, salmon populations have plummeted. The total biomass of spawning salmon in the Pacific Northwest is now estimated to be only 12–14 million kilograms. Gresh et al. [3] have calculated that the rivers of the Northwest receive just 6%–7% of the marine-derived nitrogen and phosphorus they once received from the abundant salmon population. How this shortfall may be affecting the ecology of the region's rivers or adjacent farmlands is largely unknown.

We can imagine an analogous situation developing with respect to migratory birds. Each spring, more than 30,000 tons of migratory songbirds migrate from their wintering grounds in Latin America and the Caribbean to their breeding grounds in the United States and Canada. (This biomass value is derived by combining breeding population totals from the North American Landbird Conservation Plan with species-specific weights from various sources.) If we assume these birds consume 10%–35% of their body weight per day in insects (roughly matching the requirements of a 100-gram bird and a 10-gram bird, respectively), then they are eating anywhere from 3,000–10,500 tons of insects per day. (During the breeding season, when the birds are feeding offspring, these figures would be much higher.) Several studies have shown that birds reduce insect populations in temperate forests, thus raising the question of whether ongoing declines in migratory birds pose a threat to the health of our forests and farmlands.

Similarly, one wonders how the ecology of the Serengeti would change if its migratory population of wildebeest (exceeding 1 million individuals) were to collapse, given the major role these animals surely play in terms of consuming herbaceous vegetation and redistributing nutrients via their urine and dung (Figure 1).

**Figure 1:**
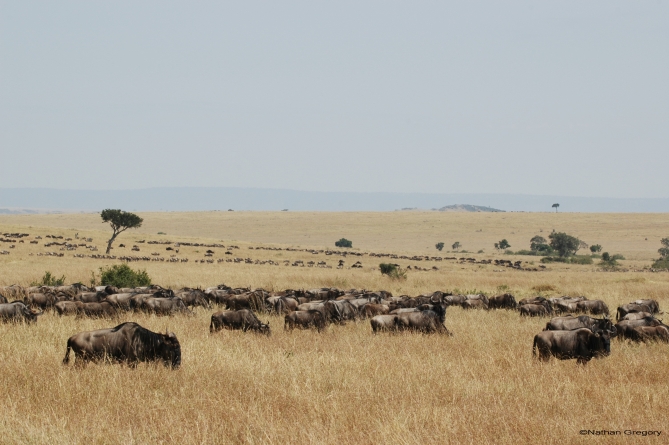
Migratory Wildebeest in the Serengeti The migration of large mammals is an increasingly threatened phenomenon around the world. (Photograph: Nathan Gregory)

## Scientific Challenges

The conservation of migratory phenomena, which entails protecting the abundance of species as opposed to merely ensuring that the animals in question do not go extinct, poses unique scientific and social challenges. On the science front, the greatest challenge may lie in understanding the demographic connectivity of migration—how events at any one stage of the migratory cycle affect other stages and the overall population trajectory of the species in question [4]. For example, the decline in breeding populations of migratory songbirds in eastern North America and Europe has attracted the attention of hundreds of scientists and generated thousands of papers over the past three decades (see summaries in [5,6]). Yet despite studies of numerous species at their breeding grounds, wintering grounds, and (to a lesser degree) stopover sites, no one can say with confidence the degree to which the observed declines are a function of the loss of breeding habitat, the loss of winter habitat, heightened mortality during migration (due to habitat destruction, pesticides, communications towers, and other factors), or some combination of the three. The answer is likely to vary from species to species and site to site, further complicating the issue. Winter storms in 1991–1992 and 1995–1996 resulted in the deaths of millions of overwintering monarch butterflies in Michoacán, Mexico, losses that were probably exacerbated by illegal logging of the forests. Yet in both cases butterfly numbers seemed to rebound within a year or two. How much winter mortality and habitat loss can the monarchs sustain before their numbers collapse and do not recover? Nobody knows.

Even for the best-studied species, the data do not yet provide a complete picture. Sillett and Holmes, for example, conducted mark-recapture studies in New Hampshire (US) and northwestern Jamaica to estimate breeding and wintering survival rates of black-throated blue warblers [7]. From these data, they deduced that over 85% of annual mortality in this species occurs during migration. Yet their estimate rests on two assumptions: first, that individuals that disappear from local study sites have died rather than moved to other breeding or wintering sites; and second, that the New Hampshire warblers winter in places where they exhibit survival rates very similar to the survival rate calculated at the Jamaican study site. Moreover, even if the 85% value is accurate, no one can say how much of an increase in migration mortality the warblers could sustain without triggering a population decline.

These sorts of questions will not be answered until scientists are able to track individual birds across a full migratory cycle, from the breeding grounds to the wintering grounds and back again, a task that requires lightweight, long-lasting satellite transmitters. Today's satellite transmitters are too heavy to place on small birds such as warblers and thrushes, but that could change in less than a decade [8].

If our knowledge of demographic connectivity in birds is fragmentary, it is nonexistent with respect to migratory bats. This lacuna seems all the more frightening in light of the many new threats (e.g., diseases, wind farms) facing bats and the growing realization of their ecological importance as pollinators and insectivores [9,10].

Almost as important as understanding demographic connectivity is knowledge of the decision rules by which migratory animals determine where to go, how long to stay, and when to leave [11]. Understanding these rules will be of tremendous value for conservation [12]. A number of researchers have expressed concern that the phenology of migration could be disrupted by climate change [13–15]. For example, the spring migration of many songbirds in both Europe and North America coincides with the leaf-out of deciduous trees and the emergence of caterpillars, which the birds eat. If, as some have theorized, the caterpillars are emerging earlier in the season due to warming temperatures, but the birds are not migrating earlier because they are relying on different cues (e.g., minor changes in day length in the tropics), then the songbirds could face serious food shortages during the migration or breeding season [16]. A firm understanding of the cues that long-distance migrants use to initiate migration could resolve whether such a scenario is truly plausible [17].

Indeed, with advances in technology, we expect the next 20 years to be a golden age in migration science, one in which scientists are finally able to answer many of the questions that have puzzled curious naturalists as far back as Aristotle. But in the meantime, the migrants continue to dwindle.

## Social and Political Challenges

Most efforts to protect biodiversity have been reactive, responding to rather than anticipating problems. Thus, species are added to the endangered species list when they are perceived to be teetering on the brink of extinction. Hotspots of biodiversity are identified based on a combination of endemicity (numbers of species found nowhere else) and threat (destruction or degradation of most of the natural vegetation in a region). But if migration is seen as a phenomenon of abundance, then its protection will require decision makers to adopt a much more proactive approach to conservation—in effect, to protect species while they are still abundant. (It may indeed have been futile to protect the last few passenger pigeons, as their very existence depended both on the abundance of conspecifics in a flock and the flocks' freedom to roam [18].) The ecology of a given species and, in particular, the nature of its migration will determine just how difficult a task conservationists face.

It seems reasonable to assume that the more jurisdictions a species crosses, the more difficult it is to protect. Thus, Swainson's thrushes flying from Canada to Brazil (via ten or more nations) would appear to pose a greater conservation challenge than salamanders trekking a few hundred yards from their breeding pond to the uplands. Yet even a relatively simple migration can pose tremendous conservation (and political) headaches. In Montana, for example, bison exit Yellowstone National Park during harsh winters. They follow established routes along the Yellowstone and Madison river valleys to lower-elevation sites where less snow cover means easier access to forage. It is a relatively short migration that falls wholly within the borders of Montana, and it is largely confined to lands managed by the federal government and the state of Montana. Yet the bison are hazed back into Yellowstone or killed when they stray outside the park due to fears that they will transmit a bacterial disease, brucellosis, to livestock. The option of removing the livestock from the winter range of the bison has not been given serious consideration.

In this issue of *PLoS Biology*, Berger et al. [2] highlight the challenges of protecting a population of migratory saiga in western Mongolia. By placing global positioning system collars on female antelopes, they were able to piece together the migratory corridor these animals followed. In particular, they identified three key bottlenecks along the route, created by natural (e.g., a lake) and anthropogenic (e.g., a town) barriers. Berger et al. predict an increasingly difficult and dangerous journey ahead for the saiga, as the region's human population increases in size and affluence, putting pressure on the migratory corridor. They further note that protecting this migration will require the participation of not only the relevant government agencies but also the local people, highlighting a crucial social dimension of conservation.

Species with a small number of stopover, breeding, or wintering sites are vulnerable to overexploitation and habitat destruction, but paradoxically, they may be easier to conserve via traditional conservation remedies than species whose migrations occur over a broad front. Thus, the eastern Pacific population of gray whales was quickly driven to the brink of extinction when 19th-century whalers discovered the shallow lagoons in Baja California, where the animals gather to mate and give birth. Subsequent protection of those sites by the Mexican government, coupled with an international ban on commercial whaling, enabled the animals to recover.

In contrast, species with large breeding or wintering ranges and diffuse migratory routes may be less vulnerable to individual threats, yet more difficult to conserve. Consider the case of the cerulean warbler, a vanishing songbird that breeds in hardwood forests across a wide swath of eastern North America and winters in montane forests from Venezuela to Peru. No one site is critical to its survival, although the bird appears to be in trouble throughout its range. Mountaintop-removal mining and development are claiming chunks of its breeding habitat; the forests where it winters are being cleared for agriculture and coca production; and its migratory route is presumably becoming increasingly inhospitable due to habitat loss as well.

It's clear that the cerulean warbler cannot be protected by creating a few reserves on its breeding and wintering grounds (especially if we do not know exactly where particular breeding populations spend the winter). On the other hand, unless its breeding and wintering habitats are protected, the species will continue to decline. There may be a few places along its migratory route where numbers of cerulean warblers can be found, but the majority of birds probably move in a rather diffuse wave between the two continents.

Thus, it is unrealistic to imagine that any discrete set of migratory reserves will work for this species. The long-term conservation of the cerulean warbler (and, we suspect, many other migrants as well) will require a broad-based, mixed strategy: creation of numerous reserves in the breeding and wintering grounds (largely by government agencies); identification and protection of key stopover sites (by government agencies and private organizations); and the use of financial incentives to encourage landowners to protect smaller patches of habitat that may be important to the warblers during their migration. (Incentives also can be used to boost the amount of breeding and wintering habitat that is protected.) Given the gaps in our knowledge of the migratory patterns and demographic connectivity of the cerulean warbler, such a strategy still amounts to a shotgun approach: protect as much habitat in as many places as possible and hope for the best. Such is the state of play for most migratory birds, bats, and insects.

The challenges—scientific, economic, and social—associated with protecting migratory species are enormous. But so too are the payoffs. We can preserve phenomena that have awed and sustained us since the dawn of humanity. We can protect ecological processes that are integral to many of the planet's ecosystems. And we can solve scientific puzzles that have baffled natural historians for millennia. If we are successful, it will be because governments and individuals have learned to act proactively and cooperatively to address environmental problems, and because we have created an international network of protected areas that is capable of sustaining much of the planet's natural diversity.

## References

[pbio-0060188-b001] Sanderson FJ, Donald PF, Pain DJ, Burfield IJ, van Bommel FPJ (2006). Long-term population declines in Afro-Palearctic migrant birds. Biol Conserv.

[pbio-0060188-b002] Berger J, Young JK, Berger KM (2008). Protecting migration corridors: Challenges and optimism for Mongolian saiga. PLoS Biol.

[pbio-0060188-b003] Gresh T, Lichatowich J, Schoonmaker P (2000). An estimation of historic and current levels of salmon production in the northeast Pacific ecosystem: evidence of a nutrient deficit in the freshwater systems of the Pacific Northwest. Fisheries.

[pbio-0060188-b004] Webster MS, Marra PP, Haig SM, Bensch S, Holmes RTI (2002). Links between worlds: Unraveling migratory connectivity. Trends Ecol Evol.

[pbio-0060188-b005] Berthold P (1993). Bird migration: A general survey.

[pbio-0060188-b006] Faaborg J (2002). Saving migrant birds: Developing strategies for the future.

[pbio-0060188-b007] Sillett TS, Holmes RT (2002). Variation in survivorship of a migratory songbird throughout its annual cycle. J Anim Ecol.

[pbio-0060188-b008] Wikelski M, Kays RW, Kasdin J, Thorup K, Smith JA (2007). Going wild—What a global small-animal tracking system could do for experimental biologists. J Exp Bio.

[pbio-0060188-b009] Nabhan GP (2004). Conserving migratory pollinators and nectar corridors in western North America.

[pbio-0060188-b010] Williams-Guillén K, Perfecto I, Vandermeer J (2008). Bats limit insects in a neotropical agroforestry system. Science.

[pbio-0060188-b011] Bauer S, Gienapp P, Madsen J (2008). The relevance of environmental conditions for departure decision changes en route in migrating geese. Ecology.

[pbio-0060188-b012] Mueller T, Olson KA, Fuller TK, Schaller GB, Murray MG (2008). In search of forage: Predicting dynamic habitats of Mongolian gazelles using satellite-based estimates of vegetation productivity. J Appl Ecol.

[pbio-0060188-b013] Coppack T, Both C (2002). Predicting life-cycle adaptation of migratory birds to global climate change. Ardea.

[pbio-0060188-b014] Jenni L, Kery M (2003). Timing of autumn bird migration under climate change: advances in long-distance migrants, delays in short-distance migrants. Proc R Soc Lond B Biol Sci.

[pbio-0060188-b015] Wilcove DS (2008). No way home: The decline of the world's great animal migrations.

[pbio-0060188-b016] Ahola M, Laaksonen T, Sippola K, Eeva T, Rainio K (2004). Variation in climate warming along the migration route uncouples arrival and breeding dates. Glob Chang Biol.

[pbio-0060188-b017] Both C, Bouwhuis S, Lessells CM, Visser ME (2006). Climate change and population declines in a long-distance migratory bird. Nature.

[pbio-0060188-b018] Schulz F (2008). Yellowstone to Yukon: Freedom to roam.

